# ﻿Micro-habitat use and seasonality of caddisfly larvae (Trichoptera) in two streams in eastern Cuba

**DOI:** 10.3897/zookeys.1263.150346

**Published:** 2025-12-10

**Authors:** Pedro López Del Castillo, Germán M. López Iborra, Liliana María Gómez Luna, Perla Alonso Eguía-Lis

**Affiliations:** 1 Centro Oriental de Ecosistemas y Biodiversidad (BIOECO), Enramada # 601, Santiago de Cuba, Cuba Centro Oriental de Ecosistemas y Biodiversidad (BIOECO) Santiago de Cuba Cuba; 2 Departamento de Ecología, IMEM Ramon Margalef, Universidad de Alicante, San Vicent del Raspeig, Spain Universidad de Alicante San Vicent del Raspeig Spain; 3 Centro Nacional de Electromagnetismo Aplicado, Universidad de Oriente, Ave. de las Américas s/n Esq. I Reparto Sueño CP 90400, Santiago de Cuba, Cuba Universidad de Oriente Santiago de Cuba Cuba; 4 Instituto Mexicano de Tecnología del Agua, Blvd. Paseo Cuauhnáhuac 8532, Progreso, 62550 Jiutepec, Morelos, Mexico Instituto Mexicano de Tecnología del Agua Morelos Mexico

**Keywords:** Assemblage composition, heterogeneity, k-means, tropical streams

## Abstract

Four microhabitats within pools (cobbles, sand, leaf litter, and bank vegetation) and one microhabitat in riffles (cobbles) were sampled in both rainy and dry seasons to identify groups of caddisfly species that share larval microhabitats across 15 sites in streams in eastern Cuba. A total of 4,367 individuals representing 13 families, 22 genera, and 36 taxa (species and morphospecies) were collected. To explore the distribution of caddisfly species by microhabitat, a k-means clustering method was used. This analysis grouped the samples into seven clusters based on species abundance across microhabitats, seasonality, altitude, and stream order. The results show that caddisfly abundance is strongly influenced by microhabitat type, seasonal variation, and river characteristics. Riffles consistently had the highest abundance of caddisflies across all sampling periods. Functional feeding groups showed variable abundance and behavior. Scrapers were strongly influenced by seasonality with high abundance in the rainy season, while generalists showed no significant seasonal effect on abundance. This study provides detailed biological trait information for specific caddisfly species, which is essential for using multimetric indices to accurately determine the health of freshwater ecosystems.

## ﻿Introduction

Caddisfly larvae are widely distributed in freshwater ecosystems, particularly in streams and rivers, where they play a crucial role in ecosystem functioning and offer several ecosystem services (Morse et al. 2019). The use of different habitats by caddisfly larvae has been widely studied (Ward 1992; Allan 1995; Urbanič et al. 2005; Martini and Waringer 2021). This group of freshwater insects exhibits diverse microhabitat use, which is influenced by ecological factors and their functional roles within aquatic ecosystems (Urbanič et al. 2005).

Microhabitat utilization by larvae is driven mainly by substrate type, water flow, and the availability of resources, which in turn affect their distribution and abundance across different stream sections (Statzner et al. 2005). Additionally, competition for food and space, predation, and prey distribution may also inﬂuence the abundance and distribution of aquatic insects (Minshall 1984). In streams, where conditions such as temperature, flow, and substrate types can vary greatly, caddisfly larvae exhibit specific behaviors in selecting microhabitats that help them optimize their survival and development (Poff and Ward 1989). The substrate is an important factor in determining habitat availability for caddisfly larvae. They can be found on rocks, woody debris, leaf litter, and submerged plants (Martini and Waringer 2021). Several key drivers, including hydrological connectivity, physicochemical factors, and life history traits influence the seasonal variation in caddisfly microhabitat use in the Neotropics. These factors interact, shaping the distribution and abundance of caddisfly larvae across different seasons (Van den Brink et al. 2013). These preferences are crucial for understanding their ecological impact and their role as bioindicators in stream environments (Brand et al. 2012).

In the Caribbean Region, the studies conducted to date have explored a range of topics, including taxonomy, natural history, distribution, and abundance of freshwater macroinvertebrates (Alonso-EguíaLis et al. 2014; Ramírez and Gutiérrez-Fonseca 2014; Bastardo and Sánchez Rosario 2017; López Del Castillo et al. 2024). However, the information about the microhabitat use, selection, and preference of microhabitats at the species level of caddisflies is sparse and scattered since this information is primarily provided in ecological remarks to the species description (Botosaneanu 1994, 2002; Flint 1996; Naranjo López and González Lazo 2005). Therefore, the main goals of this study are to describe the microhabitats used by caddisfly larvae in two mountain rivers in eastern Cuba and to identify groups of caddisfly species that share the same microhabitats. Considering the influence of seasonal changes in water flow on microhabitat availability, analyses of seasonal variation in microhabitat use have also been conducted.

## ﻿Materials and methods

### ﻿Study area

**Figure 1.**
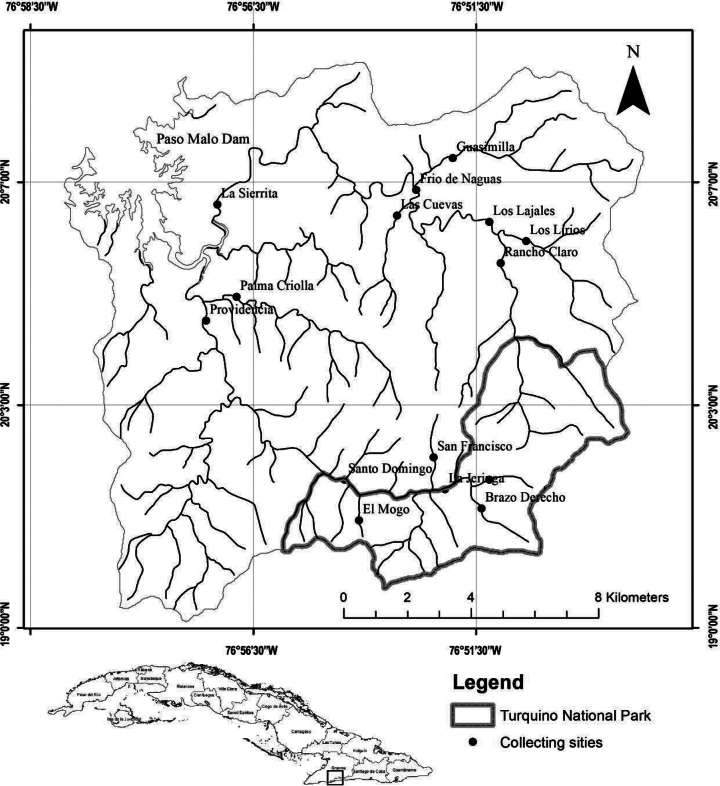
The drainage area of the Yara and Nagua river system and distribution of the sampling sites (*n* = 15).

This study was carried out in the Yara and Nagua river basins on the north slope of Sierra Maestra Mountain System, approximately 40 km southwest of Bayamo city and bounded within 19°00'00"N and 20°09'17"N and 76°58'30"W and 76°51'23"W. Both watersheds are adjacent in the headwaters, and there is a convergency of the watercourse in Paso Malo water reservoir (Fig. 1).

The geology of the watersheds is characterized by the undifferentiated presence of the El Cobre group, related to the Paleocene–Eocene period. Abundant rocks include andesites and tuffs, mixed with volcanic breccias (Núñez et al. 1989; Viña Bayés 2001). Also, ferralitic-lixiviated soil, brown without carbonates, and skeletal soils are abundant in the area (Hernández et al. 1999).

The Yara headwater is located on the northern slope of Turquino National Park. It is characterized by a small portion of tropical cloud forest, small patches of coffee plantations, and other crop areas. The predominant riparian vegetation in the Yara River basin includes montane rainforest and mesophyll evergreen forest. On the other hand, the Nagua sub-watershed has some mesophyll evergreen forest, but other types of land use are present, such as intensive agriculture areas and coffee plantations.

The Cuban archipelago experiences two distinct weather seasons each year. The period from November to April is known as the “dry season,” characterized by lower rainfall. In contrast, the “rainy season” takes place from May to October (Ortiz and Rivero 2004; Allen and Mapes 2017). The annual precipitation in the study areas ranges from 1,800 to 2,200 mm (Puentes 2001).

### ﻿Sampling

#### ﻿Collecting techniques and microhabitats

Fifteen sampling sites were established in streams from second to fourth order (Strahler 1957) in the Yara (eight sites) and Nagua rivers (seven sites). The altitude ranged from 150 to 575 m above sea level, whereas the areas of the sub-watersheds were between 0.3 and 98 km^2^ (Table 1). In the Yara River, four sites were distributed in streams of second order and the remaining four between third and fourth order. Seven points along the Nagua River were classified as third- or fourth-order streams. The only exception was Las Cuevas site, which was classified as a second-order stream (Fig. 1).

**Table 1.** Main features and location of sampling sites.

Basins	Sample sites	Latitude, Longitude	Stream order	Altitude (m)	Drainage area (km^2^)
Yara	Brazo Derecho	20°01'22"N, 76°50'49'′W	2	594	2.7
Yara	Brazo Izquierdo	20°02'08"N, 76°50'46'′W	2	558	2.5
Yara	La Jeringa	20°01'54'′N, 76°51'45'′W	3	394	8.6
Yara	San Francisco	20°02'02'′N, 76°51'53'′W	2	517	2.7
Yara	El Mogo	20°01'51'′N, 76°53'29'′W	2	637	0.3
Yara	Santo Domingo	20°02'28'′N, 76°54'09'′W	4	268	26.1
Yara	Providencia	20°04'54'′N, 76°55'57'′W	4	116	75.2
Yara	Palma Criolla	20°05'15'′N, 76°55'33'′W	3	134	14.7
Nagua	Los Lirios	20°06'11'′N, 76°50'14'′W	3	266	10.7
Nagua	Rancho Claro	20°05'48'′N, 76°50'39'′W	3	259	22.7
Nagua	Los Lajales	20°06'31'′N, 76°50'51'′W	4	231	36.6
Nagua	Frio Nagua	20°06'45'′N, 76°52'11'′W	4	168	55.1
Nagua	Guasimilla	20°07'18'′N, 76°52'01'′W	3	178	17.6
Nagua	Las Cuevas	20°06'33'′N, 76°52'33'′W	2	187	4.6
Nagua	Sierrita de Nagua	20°07'44'′N, 76°55'13'′W	4	96	98.7

The seasonality effect was accounted for by sampling in two time periods representing different river flow conditions. Two samplings were carried out in March and April 2010, which corresponds to the end of the dry season, when the river flow is at its lowest. Additional sampling was conducted in November 2010 at the onset of the dry season. However, water levels remained high during that month in the study year, as October was the month with the highest rainfall and the highest number of rainy days (INRH 2011, Fig. 2).

**Figure 2.**
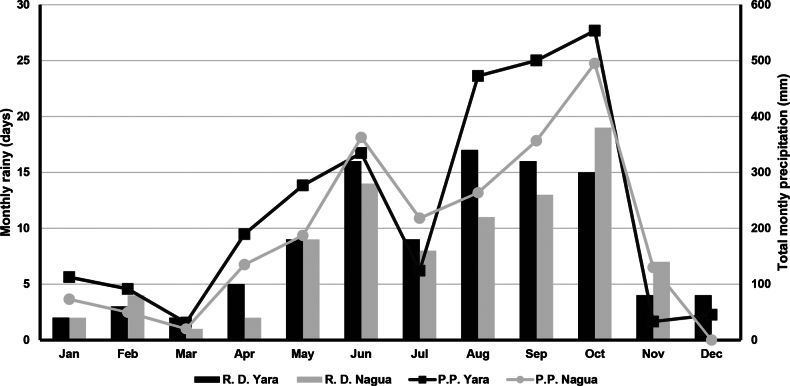
Total monthly rainy days (RD, columns, left axis) and precipitation (PP, lines, right axis) in 2010 in Yara and Nagua watersheds.

The sampling design included five microhabitats within two identified subsystems: pools and riffles. Microhabitat characterization followed the Wentworth grade scale, with modifications by Cummins (1962) and Vilenica et al. (2018), and considered substrate heterogeneity and depth, as per established methodologies (Coleman and Hynes 1970; Poole and Stewart 1976; Dudgeon 1982). Pool subsystems were the most heterogeneous, with four microhabitats—cobbles, sand, leaf litter, and riparian vegetation—present at all sites.

In the riffle subsystem, cobbles were the only microhabitat present at all sampling sites, while other microhabitats were limited and poorly represented. Sampling in the pools was carried out using four methods, one for each microhabitat: a) direct picking-up of 15 cobbles (stones) from pools with no more than 20 cm depth, b) sand was sampled using a homemade D-net (0.5 mm mesh size) by shaking the bottom in an area of 30 × 30 cm, at 20 cm depth, c) for leaf litter, a pack of 30 × 30 cm, at 10 cm deep was collected d) bank vegetation was sampled by shaking the D-net inside the submerged bank vegetation or roots systems included in a length of 3 m along the bank.

The samplings in the cobbles in riffles were made in the shallow “erosional zone”, where waters have a high flow speed, thus two complementary methods were used in this microhabitat to compensate for the loss of individuals that could be dragged by the current: a) direct picking-up out of cobbles; taking 15 cobbles with 10 cm of similar size and shape, as we did in pools b) D-Net was used to collect the caddisflies dragged by the flow when we agitated the cobbles in an area of 30 × 30 cm.

### ﻿Taxonomic identification

All samples were preserved in ethyl alcohol at 90% during the sampling until identification in the laboratory. Organisms were sorted and counted. Identiﬁcation of caddisfly larvae was made to species level with a stereomicroscope using the following keys and taxonomical description criteria: Botosaneanu and Sykora (1973), Botosaneanu (1977, 1993, 1994, 2002), Kumanski (1987), Flint (1996), Wiggins (1996, 2007). The morphospecies name corresponds to the description of Botosaneanu (1994). The specimens were preserved in 70% alcohol and stored at BIOECO’s Animal Biology Department collection after identification.

### ﻿Assignment of functional feeding groups

Specimens were assigned to functional feeding groups (FFG) based on Merritt and Cummins (1996), Cummins et al. (2005), and Ramírez and Gutiérrez Fonseca (2014) using the genus or family level to each morphospecies. The defined groups were Predators (Pr), Scrapers (Sc), Shredders (Sh-Dt shredder on plant detritus) (Sh-Hb shredder on live plant tissue), Filters (Ft), Piercers (Pc-Hb), and Collectors-Gatherers (CG).

### ﻿Data analysis

K-means clustering was used to identify groups of caddisfly species with similar use of microhabitats and seasonality (Legendre and Legendre 2012; Borcard et al. 2018). A matrix containing the total number of individuals of each species at each microhabitat in the dry (the two sampling dates averaged) and rainy seasons (20 species × 10 microhabitats-season) was built. Following Borcard et al. (2018), this matrix was transformed using the Hellinger transformation and standardized, then a k-means partitioning analysis was performed to identify the optimal number of species groups, of species according to their use of microhabitats in both seasons. The Calinski criterion was used to identify the optimal number of groups (Borcard et al. 2018). These analyses were performed with the vegan package (Oksanen et al. 2011) in R v. 4.0.3 (R Core Team 2020).

## ﻿Results

**Figure 3.**
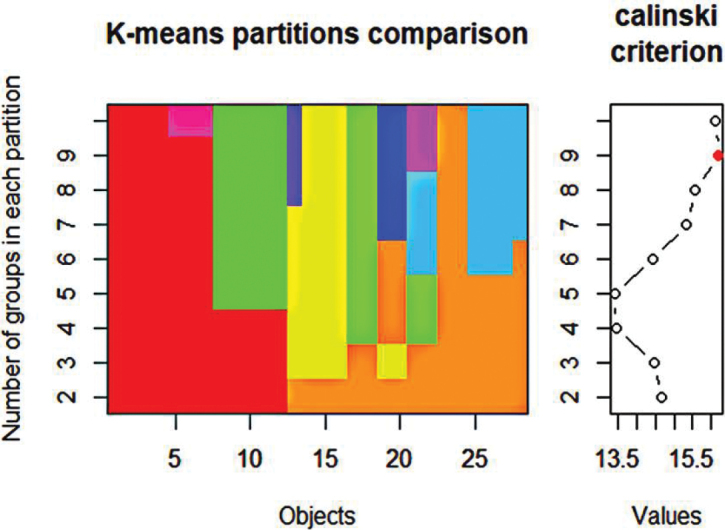
K-means cascade plot showing groups of caddisflies’ species in Yara and Nagua rivers. The left plot illustrates how the species are clustered across different k-means partitions, with the *y*-axis representing the number of groups in each partition and the *x*-axis representing the species and morphospecies. Each color denotes a distinct cluster within a given partition. The Calinski criterion posits that elevated values are indicative of more distinct clusters, and the red dot signifies the optimal cluster count. However, given the formation of single-species clusters, the number of clusters should be reduced to seven.

A total of 4,367 individuals were collected during the study, representing 13 families, 22 genera, and 36 infrageneric taxa (species and morphospecies). Of these, eight morphospecies with fewer than six specimens were excluded from the abundance analysis. This number represented 34% of the total morphospecies. Among these species were two morphospecies from the family Hydroptilidae (*Orthotrichia* sp. 1 and *Neotrichia* sp .1), and two from Helicopsychidae (*Helicopsyche cubana* (Kingsolver, 1964) and *H.* sp. (de)). Three families were represented by one morphospecies each: *Austrotinodes cubanus* (Kumanski, 1987) from Ecnomidae, *Chimarra* sp. 1 from Philopotamidae, and *Macronema* sp. 1 from Hydropsychidae. The k-means analysis revealed that the caddisfly samples clustered into seven distinct groups of species (Fig. 3) based on their abundance across microhabitats and seasonal patterns (Table 2).

**Table 2.** Grouping of species according to k-means analysis. The abundance column represents the mean number of individuals collected in the dry season and the rainy season samplings. The dominant microhabitat column identifies the microhabitat with the highest percentage of individuals averaged among species of each group in each season.

Groups	Abbreviation – Morphospecies – Functional Feeding Group (FFG). FFG: Pr = Predators, Sc = Scrapers, Sh = Shredders, (Sh-Dt shredder on plant detritus) (Sh-HbSh-Hb shredder on live plant tissue), Ft = Filters, Pc-Hb = Piercers, CG = Collectors-Gatherers.			Abundance			Dominant microhabitat use
				Dry	Rainy	Total	
I	1- Pol_sp1.	*Polycentropus* sp. 1	(Pr)	8	0	21	Generalist in dry season
	2- Hel_nea.	Helicopsyche near comosa	(Sc)	13	0		
II	3- Mar_scu.	*Marilia scudderi* Banks, 1924	(Sh)	25	4	204	Generalist in dry season
	4- Hel_sp1.	*Helicopsyche* sp. 1	(Sc)	147	28		
III	5- Hyd_sp1.	*Hydropsyche* sp.1	(Ft. Some Pr and seasonal Sc)	12	1	168	Pool stone and riffle in dry season
	6- Oxy_sp1.	*Oxyethira* sp. 1	(Pc-Hb, Sc, CG)	78	3		
	7- Och_sp1.	*Ochrotrichia* sp. 1	(Pc-Hb, Sc, CG)	7	0		
	8- Xip_cub.	*Xiphocentron cubanum* Botosaneanu, 1993	(CG)	34	12		
	9- Ant_sp1.	*Antillopsyche* sp. 1	(Pr)	11	2		
	10- Hel_cf.	Helicopsyche cf. hageni	(Sc)	9	0		
IV	11- Cer_sp1.	*Cernotina* sp1	(Pr)	10	18	423	Generalist
	12- Phy_cha.	*Phylloicus chalybeus* (Hagen, 1861)	(Sh-Dt, Sc)	62	58		
	13- Nec_cub.	*Nectopsyche cubana* (Banks, 1938)	(Sh-Hb, CG)	60	36		
	14- Hel_hag.	*Helicopsyche hageni* Banks, 1938	(Sc)	30	66		
V	15- Car_sp1.	*Cariboptila* sp. 1	(Sc)	20	88	121	Pool stones in rainy season
	16- Hel_sp2.	*Helicopsyche* sp. 2	(Sc)	0	13		
VI	17- Car_mul.	*Cariboptila mulata* Botosaneanu, 1977	(Sc)	365	1 031	2,075	Riffle in rainy season
	18- Ali_ala.	*Alisotrichia alayoana* Botosaneanu, 1977	(Sc, CG)	142	240		
	19- Ali_sp.	*Alisotrichia* sp. 1	(Sc, CG)	3	6		
	20- Ali_sp. y	*Alisotrichia* sp. y	(Sc, CG)	27	138		
	21- Leu_sp1.	*Leucotrichia* sp. 1	(Sc, CG)	1	123		
VII	22- Ato_vin.	*Atopsyche vinai* Sýkora & Botosaneanu, 1973	(Pr)	5	2	1,316	Riffle in dry season
	23- Ali_sp2.	*Alisotrichia* sp. 2	(Pc-Hb, Sc, CG)	22	2		
	24- Chi_pul.	*Chimarra pulchra* (Hagen, 1861)	(Ft)	131	0		
	25- Chi_gua.	*Chimarra guapa* Botosaneanu, 1977	(Ft)	10	0		
	26- Smi_com.	*Smicridea comma* Banks, 1924	(Ft)	267	170		
	27- Smi_sp1.	*Smicridea* sp. 1	(Ft)	35	2		
	28- Hyd_cub.	*Hydropsyche cubana* (Flint, 1962)	(Ft)	454	218		

Based on the classification of functional feeding groups (FFG) by Ramírez and Gutiérrez Fonseca (2014), the two most diverse groups were scrapers and piercers with seven species each (Table 2). In addition, scrapers had the highest abundance (1,894), followed by filterers of the family Hydroptilidae (1,159).

**Figure 4.**
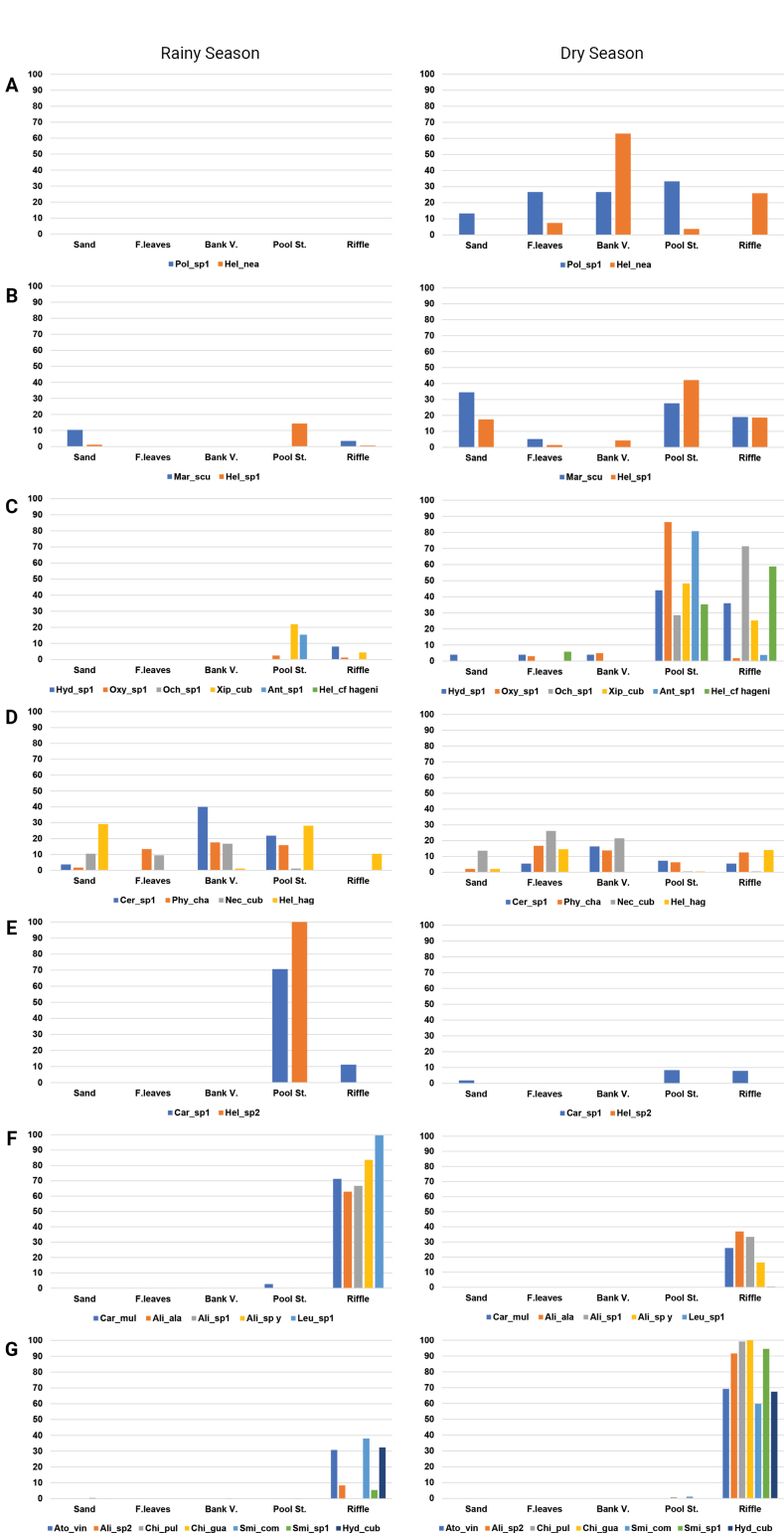
Percentage of individuals of each species (classified according to k-means groups) found in each microhabitat and season.

Group (I) (Fig. 4A) was integrated by the morphospecies *Polycentropus* sp. 1 and Helicopsyche near comosa which belong to the families Polycentropodidae and Helicopsychidae, respectively. This group reported the lowest abundance values (*n* = 21), with no individuals found in the rainy season. *Polycentropus* sp. 1 is a predator that uses all microhabitats except for the riffles. The sand microhabitat was the least used, pool stones, bank vegetation, and fallen leaves were also used with a similar proportion. On the other hand, Helicopsyche near comosa is a scraper that uses all microhabitats except sand. It is most frequently found in the bank vegetation microhabitat, followed by the riffles, among fallen leaves, and in pool stones (the last two less frequently).

Group (II) (Fig. 4B) included two species, *Marilia scudderi* (Banks 1924) (Odontoceridae) and *Helicopsyche* sp. 1 (Helicopsychidae). During the rainy season, both species presented the highest abundance values, also a generalist behavior in the use of the microhabitats. Most often, pool stones and sand were used, followed by riffles. In contrast, the microhabitats of fallen leaves and bank vegetation were used less by these two species. During the dry season, *M. scudderi*, a shredder (Ramírez and Gutiérrez Fonseca 2014), primarily inhabited sand microhabitats and a smaller number of riffles. Meanwhile, *Helicopsyche* sp. primarily used the pool stone, with fewer specimens also inhabiting the sand and riffle microhabitats.

Group III was the second most diverse group (Fig. 4C), comprising six species: *Hydropsyche* sp. 1, *Oxyethira* sp. 1, *Ochrotrichia* sp. 1, *Xiphocentron cubanum* (Botosaneanu, 1993), *Antillopsyche* sp. 1, and Helicopsyche cf. hageni (Helicopsychidae). The species in this group appear to be stone specialists, occurring both in pools and in riffles. The group also displays a diversity of FFGs, including collector-gatherers, piercers, predators, and filterers. The highest abundance was recorded during the dry season (151 individuals), mainly in the cobble microhabitat of pools. In contrast, only four species were found in the riffle microhabitat during the rainy season, with just a few individuals (18). The most abundant species were *Oxyethira* sp. (81 individuals) and *X. cubanum* (46) in the pool cobble microhabitats.

Group (IV) was the third most abundant (Fig. 4D), comprising *Cernotina* sp. 1, *Phylloicus chalybeus*, *Nectopsyche cubana*, and *Helicopsyche hageni*. These species belong to the families Polycentropodidae, Calamoceratidae, Leptoceridae, and Helicopsychidae, respectively. This group showed generalist behavior in microhabitat use and each species belongs to a different functional feeding group. Seasonality did not significantly affect their abundance as values were similar in both the dry and rainy seasons. The only notable exception was *Nectopsyche cubana*, whose abundance decreased by nearly 50% from the dry to the rainy season.

Group (V) (Fig. 4E) contained *Cariboptila* sp. 1 and *Helicopsyche* sp. 2 from the families Glossosomatidae and Helicopsychidae, respectively. These morphospecies are both grouped as scrappers FFG, and their preferred microhabitat were pool stones. *Helicopsyche* sp. 2 was only present in the rainy season. During the rainy season, *Cariboptila* sp. 1 mainly used the microhabitats of pool stones and riffles, and in the dry season, it also used sand but with fewer specimens.

Group (VI) had the highest abundance among all the groups in the k-means analysis (Fig. 4F), formed by *Cariboptila mulata*, *Alisotrichia alayoana*, *Alisotrichia* sp. 1, *Alisotrichia* sp. y, and *Leucotrichia* sp. 1. They are members of the Glossosomatidae and Hydroptilidae. The riffles were the preferred microhabitats of this group species. Meanwhile, all members of the group were scrapers. Moreover, in the rainy season, abundance values were twice as high compared to the dry season. *Cariboptila mulata* was the most abundant species, representing 67% of the group.

Seven species were present in Group (VII) (Fig. 4G), the most diverse in our analysis. The species included *Atopsyche vinai*, *Alisotrichia* sp. 2, *Chimarra pulchra*, *C. guapa*, *Smicridea comma*, *Smicridea* sp. 1, and *Hydropsyche cubana*. They are members of the Hydrobiosidae, Philopotamidae and Hydropsychidae. During the dry season, they preferred almost exclusively the riffle microhabitat. Filterers dominated the functional feeding groups, but predators and scrapers were also present. In the dry season, abundance was higher by 70% over abundance in the rainy season, with *H. cubana* and *S. comma* being the most abundant species in both seasons (Table 2) within the group.

## ﻿Discussion

Some caddisfly species show the effect of seasonality on their abundances (Extence 1981; Baptista et al. 2001; Reyes Torres and Ramírez 2018). This study demonstrates that there are groups of caddisfly morphospecies that significantly differ in the use of microhabitats and seasonality between rivers. Also, there are groups of species with a clear use of a specific microhabitat. For example, the larvae of the genus *Xiphocentron cubanum* and *Antillopsyche* sp. 1 showed a clear association with pool stone microhabitats accumulated on the stream bed. Also, species belonging to the Hydropsychidae and Glossosomatidae were predominantly found in riffles. A clear seasonality was observed, since some species used specific microhabitats exclusively during the rainy or dry season. On the other hand, these groups obtained by the k-means are related to the FFG. These groups had the most diversity where species used different microhabitats or were generalist, such as Groups (III) and (IV). The study found that scrapers and collectors typically inhabited riffles. In these conditions, the turbulent water in riffles prevents the accumulation of fine sediments and organic matter, keeping rock surfaces clean and well oxygenated. Additionally, riffle microhabitats received more light due to their shallow depth and clearer substrates. In contrast, predators tended to inhabit pools. For example, polycentropodids construct fixed retreats and capture nets that function most effectively in areas with low to moderate flow, where drifting prey is available, but the current is not strong enough to destroy their nets (Chará Serna et al. 2010; Brand et al. 2012; Ramírez and Gutiérrez Fonseca 2014).

Group (I) includes the species *Polycentropus* sp. 1 and Helicopsyche near comosa. Both speciesshowed a strong relationship between seasonality and their presence because they were only present during the rainy season. A dry period may directly or indirectly affect species and the community (Lake 2003). These species were generalist, while *Polycentropus* sp. 1 was found mainly in the pool microhabitat. Comparable abundance patterns were observed in Bayamesa National Park (López Del Castillo et al. 2004) and in the Cauto watershed (Deler Hernández et al. 2007) offer support of our findings. However, Helicopsyche near comosa is a scraper that used mostly bank vegetation and riffle microhabitats. Also, this species showed little use of packed leaves and pool stones and avoided sandy microhabitats. Scrapers are associated mainly with the flowing water (Merritt and Cummins 1996; Ramírez and Gutiérrez Fonseca 2014).

Group (II) species, *Marilia scudderi* and *Helicopsyche* sp. 1, are generalists in their microhabitat uses. Also, due to their high abundance in the dry season, these species probably are influenced by the seasons. In Bayamesa National Park, *M. scudderi* was collected in rivers with similar conditions to this study (López Del Castillo et al. 2004), but few specimens were found using riffles and pool habitats. The genus *Marilia* has been reported as a predator and collector-gatherer in southeastern Brazil (Froehlich and Oliveira 1997; Oliveira and Nessimian 2010). Similarly, Chará Serna et al. (2010) described *Marilia* species in Colombia as shredders. Reynaga (2009) reported the genus in the collector-gatherer functional group, while Tomanova et al. (2006) described it as a scraper and collector-gatherer. Reynaga and Rueda Martín (2014) recommended not to assign a functional feeding group to the genus *Marilia*. The morphospecies *Helicopsyche* sp. was observed in all samples using mainly the microhabitats of pool stones and sands. The primary source of food for this species is algae and associated material on stones (Cummins 1973).

The second most diverse group is Group III, which primarily utilized the microhabitats of pool stones and riffles. Additionally, specimens in this group have five different feeding strategies (Table 2), the highest among the analyzed groups. Urbanič et al. (2005) found similar results where the major feeding types were found in marginal habitats. In the dry season, *Oxyethira* sp. 1 was the most abundant morphospecies, with 78 specimens. In the Cuban archipelago, this genus has a wide distribution (Naranjo López and González Lazo 2005). A similar result was obtained in the Mayari River in northeast Cuba; meanwhile, in the Yara River, the abundance was similar in both seasons (López Del Castillo et al. 2007). Although both *Oxyethira* sp. 1 and *Ochrotrichia* sp. 1 are classified as filter feeders, their distinct patterns of microhabitat use suggest niche differentiation, which may reduce interspecific competition and support the overall diversity of Hydroptilidae in these streams (Huryn 2009; Holzenthal et al. 2015). The species *Xiphocentron cubanum* has three subspecies distributed in three separate regions of Cuba (Botosaneanu 1993). It is also a species adapted to clean water with a score of 9 out of 10 in the BMWP/Cub index (Alonso-EguíaLis et al. 2014). *Antillopsyche* sp. 1 is a Greater Antillean endemic genus (Chamorro and Holzenthal 2011) and a predator belonging to the Polycentropodidae (Ramírez and Gutiérrez Fonseca 2014). Henriques Oliveira and Nessimian (2010) obtained similar results, finding that some morphospecies of Polycentropodidae used the microhabitat litter in the pool, while other morphospecies used the gravel.

Group III also exhibited marked seasonal variation, with a significantly higher abundance of Helicopsyche cf. hageni and *Antillopsyche* sp. 1 during the dry season compared to the rainy season. During the dry season, reduced water flow likely leads to greater habitat stability and increased availability of preferred microhabitats such as pool stones and riffles, which can support higher larval densities (Lake 2003). Additionally, lower water levels may concentrate resources and reduce drift, making conditions more favorable for these species. The high abundance of Helicopsyche cf. hageni and *Antillopsyche* sp. 1 in the dry season could also reflect their life cycle synchronization with periods of lower flow, which may enhance survival and growth rates. These findings highlight the importance of seasonal hydrological changes in shaping the composition and structure of caddisfly assemblages in tropical streams.

Within streams, habitat generalists occupy a wide variety of habitat types within the river (Saito and Tojo 2016); this pattern is consistent with the morphospecies of Group (IV). The abundances of each morphospecies were similar in both seasons. Although these species are generalist by the habitats they use, they preferred microhabitats in the pools with low water flows and avoided the currents during the rainy season. The most abundant species was *Phylloicus chalybeus*, with 120 specimens (total of both seasons). Species of this genus are abundant in pools, mainly in streams of low order with riparian vegetation (Springer 2010). They are shredders, which play a critical role in leaf pack processing, food chains, and energy fluxes that contribute directly to the river continuum (Vannote et al. 1980; Reyes Torres and Ramírez 2018). Similar results with *Phylloicus chalybeus* were obtained for Yara River, whereas Mayari River showed considerably more abundance in the dry season (López Del Castillo et al. 2007).

Also in Group IV, *Nectopsyche cubana* is a shredder of living aquatic macrophyte tissue. It can also be a collector-gatherer, in which case bacteria and meiofauna are the primary sources of nutrition (Huryn 2009). Due to seasonality, this species had its abundance highest at two times during the dry season, a result obtained by López Del Castillo et al. (2007) in the Yara River. In this period, the pool microhabitats have more stability and availability since they are less affected by the currents (Baptista et al. 2001).

The morphospecies with the lowest abundance in this group was *Cernotina* sp. 1, which does not appear to be seasonal but prefers both bank vegetation and pool-stone microhabitats. *Cernotina* is a Neotropical genus (Chamorro 2003) and a predator according to Ramírez and Gutiérrez Fonseca (2014), which uses silken nets to capture their invertebrate prey (Holzenthal et al. 2015). Previous reports of this genus in Bayamesa National Park and Mayari River showed a low number of specimens (López Del Castillo et al. 2004; López Del Castillo et al. 2007).

Group (V) contains two scrapper morphospecies, which are highly influenced by seasonality and preferentially inhabited the pool stones microhabitat during the rainy season. The stream flow regime is one of the most important factors that determines physical, chemical, and biological processes (Allan and Castillo 2007), influencing substrate stability, suitability, and the aquatic environment composition. During the rainy season, the increase in water level allowed more availability of habitats for this group. These results obtained with *Cariboptila* sp .1 are consistent with those obtained in the Yara River, but the opposite in the Mayari River (López Del Castillo et al. 2007).

In this study, the highest abundance value was found in Group (VI) (*n* = 2,075). Because many species have three times more specimens in the rainy season than during the dry season, the specimens in this group presented a high influence of seasonality. All the species in this group belong to the scrapper FFG. Scrappers are important in streams and lakes because they consume the biofilm formed by diatoms, algae, and cyanobacteria, contributing to the food chain (Huryn 2009). Also, this FFG is very conspicuous, increasing the level of secondary productivity and, therefore, controlling the productivity and biomass of the epilithic communities (Jin and Ward 2007). Detritus and algae are transported in the runoff during the rainy season, stimulating biological communities and allowing species with short life cycles to reestablish (Fisher 1983). The species *Cariboptila mulata*, with a distribution in the oriental area of the Cuban archipelago (Naranjo López and González Lazo 2005), was the most abundant in the study (*n* = 1,396), even though in previous studies, this species had low abundance values (López Del Castillo et al. 2004, 2007). The other four species in this group also belong to the Hydroptilidae. These morphospecies can change their way of feeding and, for this reason, could be assigned to more than one FFG (Ramírez and Gutiérrez Fonseca 2014). The abundance of this family and their use of riffles have already been reported elsewhere (López Del Castillo et al. 2004; Naranjo López and González Lazo 2005).

The group with the highest diversity and second-highest abundance in this study is Group (VII), also with a clear seasonal influence, presenting a remarkably high abundance during the dry season. Seven species were present in the more diverse Group (VII) (Fig. 3G), including *Atopsyche vinai*, *Alisotrichia* sp. 2. *Chimarra pulchra*, *C. guapa*, *Smicridea comma*, *Smicridea* sp. 1, and *Hydropsyche cubana*. They are members of the Hydrobiosidae, Philopotamidae, and Hydropsychidae, respectively. During both seasons, they preferred the riffle microhabitat. Filterers dominated the FFG, but predators and scrapers were also present. There was greater abundance in the dry season, 70% higher compared to the rainy season.

The events associated with high and low flow regimes habitually serve as ecological “bottlenecks”, allowing stresses and also opportunities for several freshwater species (Poff and Ward 1989). Also, the high abundance value during the dry season could be due to the reduced availability of habitat, consequently, insects may concentrate in these limited areas (Dudgeon 1997). Substrates are used by species with similar morphological and functional characteristics, which allow them to live in these habitats (Oliveira and Nessimian 2010), as the filtering FFG in riffle microhabitats. Five of the seven species are filterers, and within this group, there are two highly abundant species, *Smicridea comma* and *Hydropsyche cubana*. Similar results in England were obtained by Extence (1981) due to the reduction in water depth, which favors the filtering species *Hydropsyche angustipennis*.

This study will provide precision in the applicability of monitoring programs in Cuba and elsewhere in the Greater Antilles. Also, information related to some biological traits of some species is provided. Further studies in the region should focus on expanding the biological trait database for caddisfly species and exploring the effects of environmental changes on their assemblages. Such research will be essential for developing robust multimetric indices for stream health assessment in the region.

## ﻿Additional information

### Use of AI

No use of AI was reported.

### Author ORCIDs

Pedro López Del Castillo https://orcid.org/0000-0002-0892-3278

Germán M. López Iborra https://orcid.org/0000-0003-3045-5498

Liliana María Gómez Luna https://orcid.org/0000-0002-1282-3392

Perla Alonso Eguía-Lis https://orcid.org/0000-0002-0802-2461

### Data availability

All of the data that support the findings of this study are available in the main text.

## References

[B1] Allan JD (1995) Stream Ecology. Structure and Function of Running Waters. Chapman & Hall, London, 388 pp.

[B2] Allan JD Castillo MM (2007) Stream Ecology: Structure and Function of Running Waters. Springer Science & Business Media, Dordrecht, 436 pp.

[B3] Allen TL Mapes BE (2017) The late spring Caribbean rain‐belt: Climatology and dynamics. International Journal of Climatology 37(15): 4981–4993. 10.1002/joc.5136

[B4] Alonso-EguíaLis PE Mora JM Campbell B Springer M (2014) Diversidad, Conservación y uso de los Macroinvertebrados Dulceacuícolas de México, Centroamérica, Colombia, Cuba y Puerto Rico. Instituto Mexicano de Tecnología del Agua, Mexico, 442 pp.

[B5] Banks N (1924) Descriptions of new neuropteroid insects. Bulletin of the Museum of Comparative Zoology (Harvard) 65(12): 421–455.

[B6] Banks N (1938) New West Indian neuropteroid insects. Revista de Etologia 9: 285–304.

[B7] Baptista DF Buss DF Dorvillé FM Nessimian JL (2001) Diversity and habitat preference of aquatic insects along the longitudinal gradient of the Macaé river basin, Rio de Janeiro, Brazil. Revista Brasileira de Biologia 61(2): 249–258. 10.1590/S0034-71082001000200007 11514892

[B8] Bastardo RH Sánchez Rosario A (2017) State of knowledge of aquatic macroinvertebrates of Hispaniola island. Actualidades Biologicas 39: 75–81.

[B9] Borcard D Gillet F Legendre P (2018) Numerical Ecology with R. Springer, Cham, 299–367. 10.1007/978-3-319-71404-2

[B10] Botosaneanu L (1977) Trichoptéres (imagos) de Cuba captures par moi-meme in 1973 (Insecta, Trichoptera). Fragmenta Entomologica 13: 231–284.

[B11] Botosaneanu L (1993) Notes on the Cuban *Xiphocentron* (Trichopetera: Xiphocentronidae). Entomology Zoology 103: 293–298.

[B12] Botosaneanu L (1994) A study of the larvae of caddisflies (Trichoptera) from Cuba. Tropical Zoology 7(2): 451–475. 10.1080/03946975.1994.10539267

[B13] Botosaneanu L (2002) An annotated checklist of caddisflies from the Caribbean islands, with distribution and bibliography (Insecta, Trichoptera). Bulletin de la Société Entomologique de France 107(1): 79–108. 10.3406/bsef.2002.16821

[B14] Botosaneanu L Sykora J (1973) Sur quelques trichoptères (Insecta: Trichoptera) de Cuba. Résultats des Expéditions Biospéologiques cubano-roumaines à Cuba 1: 379–407.

[B15] Brand C Miserendino ML Epele LB (2012) Spatial and temporal pattern of caddisfly distribution at a mesohabitat scale in two Patagonian mountain streams subjected to pastoral use. International Review of Hydrobiology 97(2): 83–99. 10.1002/iroh.201111368

[B16] Chamorro ML (2003) Seven new species of Polycentropodidae (Trichoptera) from Nicaragua and Costa Rica. Proceedings of the Entomological Society of Washington 105(2): 484–498.

[B17] Chamorro ML Holzenthal RW (2011) Phylogeny of Polycentropodidae Ulmer, 1903 (Trichoptera: Annulipalpia: Psychomyioidea) inferred from larval, pupal and adult characters. Invertebrate Systematics 25(3): 219–253. 10.1071/IS10024

[B18] Chará Serna AM Chará JD Zúñiga MC Pedraza GX Giraldo LP (2010) Clasificación trófica de insectos acuáticos en ocho quebradas protegidas de la ecorregión cafetera colombiana. Universitas Scientiarum 15(1): 27–36. 10.11144/javeriana.SC15-1.tcoa

[B19] Coleman MJ Hynes HBN (1970) The vertical distribution of the invertebrate fauna in the bed of a stream. Limnology and Oceanography 15(1): 31–40. 10.4319/lo.1970.15.1.0031

[B20] Cummins RW (1962) An evaluation of some techniques for the collection and analysis of benthinc samples with special emphasis on lotic waters. American Midland Naturalist 67(2): 477–504. 10.2307/2422722

[B21] Cummins KW (1973) Trophic relations of aquatic insects. Annual Review of Entomology 18(1): 183–206. 10.1146/annurev.en.18.010173.001151

[B22] Cummins KW Merritt RW Andrade PC (2005) The use of invertebrate functional groups to characterize ecosystem attributes in selected streams and rivers in south Brazil. Studies on Neotropical Fauna and Environment 40(1): 69–89. 10.1080/01650520400025720

[B23] Deler Hernández A Megna YS González Lazo DD Cracasés Torres CN (2007) Insectos acuáticos y áreas prioritarias para la conservación en la cuenca alta del Río Cauto (Santiago de Cuba, Cuba). Boletin de la SEA 40: 451–461.

[B24] Dudgeon D (1982) Aspects of the microdistribution of insect macrobenthos in a forest stream in Hong Kong. Archiv für Hydrobiologie (Supplement 64): 221–239.

[B25] Dudgeon D (1997) Life histories, secondary production and microdistribution of hydropsychid caddisflies (Trichoptera) in a tropical forest stream. Journal of Zoology (London, England) 243(1): 191–210. 10.1111/j.1469-7998.1997.tb05763.x

[B26] Extence CA (1981) The effect of drought on benthic invertebrate communities in a lowland river. Hydrobiologia 83(2): 217–224. 10.1007/BF00008269

[B27] Fisher SG (1983) Succession in streams. In: Barnes JR Minshall GW (Eds) Stream Ecology: Application and Testing of General Ecological Theory.Plenum Press, New York, 7–27. 10.1007/978-1-4613-3775-1_2

[B28] Flint Jr OS (1996) Checklist of the Trichoptera, caddisflies, of Cuba. Cocuyo 5: 15–17.

[B29] Froehlich CG Oliveira LG (1997) Ephemeroptera and Plecoptera nymphs from riffles in low-order streamis in southeastern Brazil. In: Landolt P Sartori M (Eds) Ephemeroptera & Plecoptera: Biology–Ecology–Systematics.MTL, Fribourg, 180–185.

[B30] Hagen H (1861) Synopsis of the Neurptera of North America. Smithsonian Miscellaneous Collections 4: 347.

[B31] Henriques Oliveira AL Nessimian JL (2010) Aquatic macroinvertebrate diversity and composition in streams along an altitudinal gradient in Southeastern Brazil. Biota Neotropica 10(3): 115–128. 10.1590/S1676-06032010000300012

[B32] Hernández A Pérez PJ Bosh D Rivero L (1999) Nueva versión de clasificación genética de los suelos de Cuba. Instituto de Suelos, La Habana, Cuba.

[B33] Holzenthal RW Thomson RE Ríos Touma B (2015) Order Trichoptera. In: Thorp JH Rogers DC (Eds) Thorp and Covich’s Freshwater Invertebrates.Academic Press, San Diego, 965–1002. 10.1016/B978-0-12-385026-3.00038-3

[B34] Huryn AD (2009) Aquatic Insects Ecology, Feeding, and Life History. University of Alabama, Tuscaloosa, 132–143. 10.1016/B978-012370626-3.00159-9

[B35] INRH (2011) Delegación Provincial de Recursos Hidráulicos Granma. Reporte 2010.

[B36] Jin HS Ward GM (2007) Life history and secondary production of *Glossosoma nigrior* Banks (Trichoptera: Glossosomatidae) in two Alabama streams with different geology. Hydrobiologia 575(1): 245–258. 10.1007/s10750-006-0370-2

[B37] Kingsolver JM (1964) New species of Trichoptera from Cuba. Proceedings of the Entomological Society of Washington 66(4): 257–257.

[B38] Kumanski KP (1987) On caddisflies (Trichoptera) of Cuba. Acta Zoologica Bulgarica 34: 3–35.

[B39] Lake PS (2003) Ecological effects of perturbation by drought in flowing waters. Freshwater Biology 48(7): 1161–1172. 10.1046/j.1365-2427.2003.01086.x

[B40] Legendre P Legendre L (2012) Numerical Ecology (3^rd^ edn). Elsevier Science BV, Amsterdam, 1006 pp.

[B41] López Del Castillo P Naranjo López JC González Lazo DD Trapero A Fernández JL Pérez J (2004) Insectos acuáticos del Parque Nacional “La Bayamesa”, Cuba. Boletin de la SEA 35: 225–231.

[B42] López Del Castillo P Naranjo López JC Fernández Triana JL González Lazo DD (2007) Caddisflies (Insecta: Trichoptera) in two rivers of eastern Cuba. In: Proceedings of the 12^th^ International Symposium on Trichoptera. The Caddis Press, Columbus, Ohio, 169–173.

[B43] López Del Castillo P Gómez Luna LM López Iborra GM (2024) Microhabitat use and seasonality of mayflies (Ephemeroptera) in two streams in eastern Cuba. Aquatic Insects 45(3): 384–401. 10.1080/01650424.2023.2299810

[B44] Martini J Waringer J (2021) Dynamic microhabitat shifts in space and time of caddisfly larvae (Insecta: Trichoptera) in a first‐order calcareous mountain stream. Biologia 76(9): 2527–2541. 10.1007/s11756-021-00741-w

[B45] Merritt RW Cummins KW [Eds] (1996) An Introduction to the Aquatic Insects of North America. Kendall Hunt, Dubuque, 862 pp.

[B46] Minshall GW (1984) Aquatic insect–substratum relationships. In: Resh VH Rosenberg DM (Eds) The Ecology of Aquatic Insects.Praeger Scientific, New York, 358–400.

[B47] Morse JC Frandsen PB Wolfram G Thomas JA (2019) Diversity and ecosystem services of Trichoptera. Insects 10(5): 125. 10.3390/insects10050125 31052441 PMC6572163

[B48] Naranjo López JC González Lazo DD (2005) Orden Trichoptera en Cuba. Boletin de la SEA 1(36): 147–152.

[B49] Núñez A Viña Bayes N Graña A (1989) Regiones naturales antrópicas. In: Hernández JR, Sánchez Herrero A, Propín E, Buznego E, Lorenzo AC, Mon M, Azcue A, et al. (Eds) Nuevo Atlas Nacional de Cuba. Instituto de Geografía, La Habana & Madrid, 232 pp.

[B50] Oksanen J Blanchet F Kindt R Legendre P O’Hara R Simpson G (2011) Vegan: community ecology package (version 1.17-11). https://cran.r-project.org/web/packages/vegan/index.html

[B51] Oliveira ALHD Nessimian JL (2010) Spatial distribution and functional feeding groups of aquatic insect communities in Serra da Bocaina streams, southeastern Brazil. Acta Limnologica Brasiliensia 22(4): 424–441. 10.4322/actalb.2011.007

[B52] Ortiz BP Rivero A (2004) Índices climáticos para la determinación y simulación de las señales de la variabilidad climática en diferentes escalas espacio temporales. Revista Cubana de Meteorología 11(1): 65–75.

[B53] Poff NL Ward JV (1989) Implications of streamflow variability and predictability for lotic community structure: A regional analysis of streamflow patterns. Canadian Journal of Fisheries and Aquatic Sciences 46(10): 1805–1818. 10.1139/f89-228

[B54] Poole WC Stewart KW (1976) The vertical distribution of macrobenthos within the substratum of the Brazos River, Texas. Hydrobiologia 50(2): 151–160. 10.1007/BF00019818

[B55] Puentes G (2001) Regionalización climática de los macizos montañosos orientales. In: Viña N Fong A Maceira D (Eds) Diversidad Biológica de los Macizos Montañosos Orientales.Centro Oriental de Ecosistemas y Biodiversidad (BIOECO), Santiago de Cuba, 14–25.

[B56] R Core Team (2020) R: a language and environment for statistical computing. RFS Computing, Vienna, Austria.

[B57] Ramírez A Gutiérrez-Fonseca PE (2014) Functional feeding groups of aquatic insect families in Latin America: A critical analysis and review of existing literature. Revista de Biología Tropical 62(2): 155–167. 10.15517/rbt.v62i0.15785 25189076

[B58] Reyes Torres LJ Ramírez A (2018) Life history and phenology of *Phylloicus pulchrus* (Trichoptera: Calamoceratidae) in a tropical rainforest stream of Puerto Rico. Revista de Biología Tropical 66(2): 814–825. 10.15517/rbt.v66i2.33411

[B59] Reynaga MC (2009) Hábitos alimentarios de larvas de Trichoptera (Insecta) de una cuenca subtropical. Ecología Austral 19(3): 207–214.

[B60] Reynaga MC Rueda Martín PA (2014) Trophic analysis of three species of *Marilia* (Trichoptera: Odontoceridae) from the neotropics. Revista de Biología Tropical 62(2): 543–550. 10.15517/rbt.v62i2.9959 25102638

[B61] Saito R Tojo K (2016) Comparing spatial patterns of population density, biomass, and genetic diversity patterns of the habitat generalist mayfly *Isonychia japonica* Ulmer (Ephemeroptera: Isonychiidae) in the Chikuma–Shinano river basin. Freshwater Science 35(2): 724–737. 10.1086/686537

[B62] Springer M (2010) Macroinvertebrados de agua dulce de Costa Rica I, capítulo 7: Trichoptera. Revista de Biología Tropical 58(4): 151–181. 22043666

[B63] Statzner B rigoux SM Leichtfried M (2005) Mineral grains in caddisfly pupal cases and streambed sediments: Resource use and its limitation through conflicting resource requirements. Limnology and Oceanography 50(2): 713–721. 10.4319/lo.2005.50.2.0713

[B64] Strahler AN (1957) Quantitative analysis of watershed geomorphology. Eos, Transactions American Geophysical Union 38(6): 913–920. 10.1029/TR038i006p00913

[B65] Tomanova S Goitia E Helesic J (2006) Trophic levels and functional feeding groups of macroinvertebrates in Neotropical streams. Hydrobiologia 556(1): 251–264. 10.1007/s10750-005-1255-5

[B66] Urbanič G Toman MJ Krušnik C (2005) Microhabitat type selection of caddisfly larvae (Insecta: Trichoptera) in a shallow lowland stream. Hydrobiologia 541(1): 1–12. 10.1007/s10750-004-4314-4

[B67] Van den Brink FWB Van der Velde G Wijnhoven S (2013) Seasonal changes in caddis larvae assemblages in river-floodplain habitats along a hydrological connectivity gradient. Hydrobiologia 716(1): 75–85. 10.1007/s10750-013-1545-2

[B68] Vannote RL Minshall GW Cummins KW Sedell JR Cushing CE (1980) The river continuum concept. Canadian Journal of Fisheries and Aquatic Sciences 37(1): 130–137. 10.1139/f80-017

[B69] Vilenica M Brigić A Sartori M Mihaljević Z (2018) Microhabitat selection and distribution of functional feeding groups of mayfly larvae (Ephemeroptera) in lotic karst Habitats. Knowledge and Management of Aquatic Ecosystems 419(17): 1–12. 10.1051/kmae/2018011

[B70] Viña Bayés N (2001) Caracterización geográfica. In: Fong A Viña N Maceira D (Eds) Diversidad Biológica de los Macizos Montañosos Orientales.Centro Oriental de Ecosistemas y Biodiversidad (BIOECO), Santiago de Cuba, 4–12.

[B71] Ward JV (1992) Aquatic Insect Ecology. 1. Biology and Habitat. John Wiley y Sons, _New York 438 pp.

[B72] Wiggins GB (1996) Larvae of the North American Caddisﬂy Genera (Trichoptera), 2^nd^ edn. University of Toronto Press, Toronto, 457 pp. 10.3138/9781442623606

[B73] Wiggins GB (2007) Caddisflies: Architects under water. American Entomologist 53(2): 78–85. 10.1093/ae/53.2.78

